# Supplementary figures and images for: T Regulatory CD4+CD25+FoxP3+ Lymphocytes in the Peripheral Blood of Left-Sided Colorectal Cancer Patients
Source: Medicina (Kaunas). 2019 Jun 25;55(6):307. doi: 10.3390/medicina55060307 (PMC6631385; doi:10.3390/medicina55060307)

**A**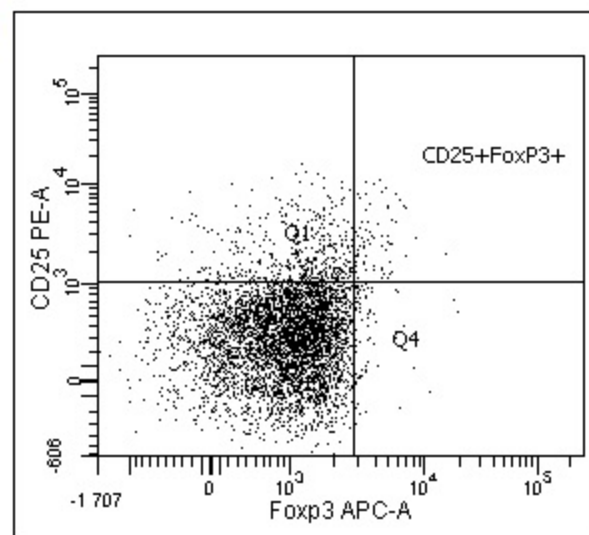**B**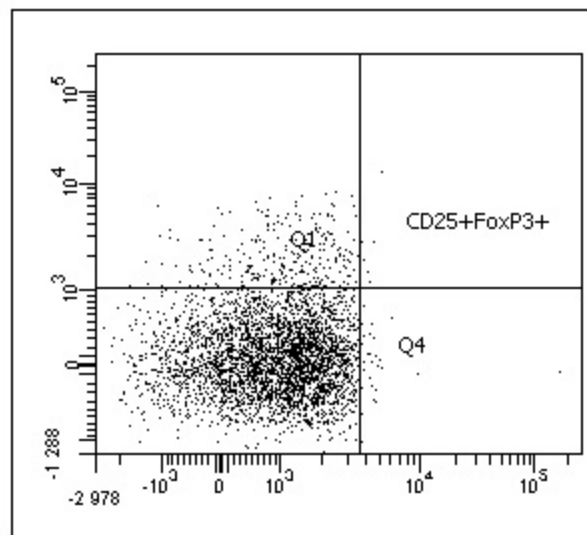**C**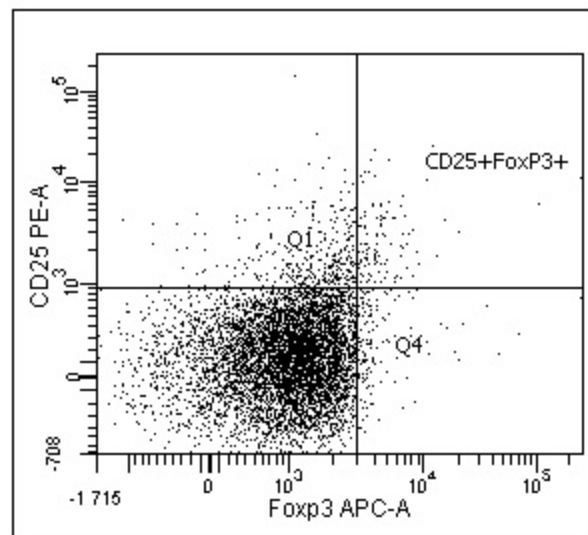**D**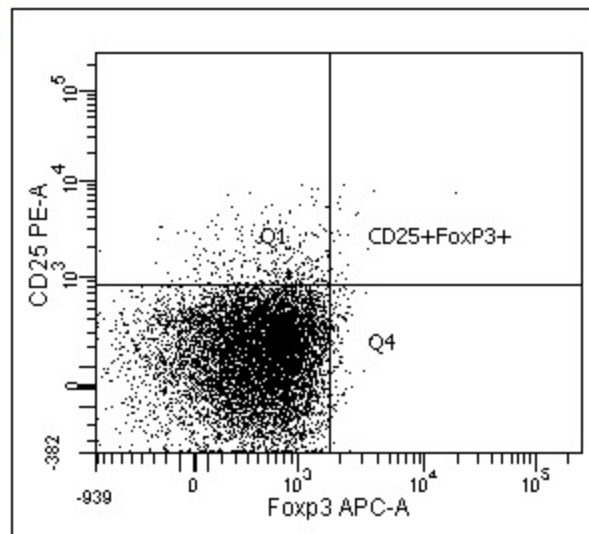**E**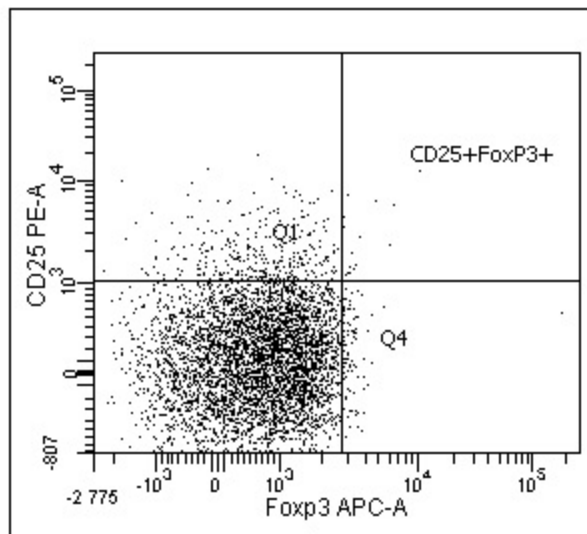

Supplement: Supplementary file 1 [file medicina-55-00307-s001.pdf]
